# Culture and detection of primary cilia in endothelial cell models

**DOI:** 10.1186/s13630-015-0020-2

**Published:** 2015-09-30

**Authors:** Yi Chung Lim, Sue R. McGlashan, Michael T. Cooling, David S. Long

**Affiliations:** Auckland Bioengineering Institute, University of Auckland, 70 Symonds Street, Auckland, 1142 New Zealand; Department of Anatomy with Radiology, University of Auckland, 85 Park Road, Auckland, New Zealand; Department of Engineering Science, University of Auckland, 70 Symonds Street, Auckland, New Zealand

**Keywords:** Primary cilium, Foetal bovine serum, Serum starvation, HUVEC, HMEC-1, Cilia incidence, Cobblestone morphology

## Abstract

**Background:**

The primary cilium is a sensor of blood-induced forces in endothelial cells (ECs). Studies that have examined EC primary cilia have reported a wide range of cilia incidence (percentage of ciliated cells). We hypothesise that this variation is due to the diversity in culture conditions in which the cells are grown. We studied two EC types: human umbilical vein endothelial cells (HUVECs) and human microvascular endothelial cells (HMEC-1s). Both cell types were grown in media containing foetal bovine serum (FBS) at high (20 % FBS and 10 % FBS for HUVECs and HMEC-1s, respectively) or low (2 % FBS) concentrations. Cells were then either fixed at confluence, serum-starved or grown post-confluence for 5 days in corresponding expansion media (cobblestone treatment). For each culture condition, we quantified cilia incidence and length.

**Results:**

HUVEC ciliogenesis is dependent on serum concentration during the growth phase; low serum (2 % FBS) HUVECs were not ciliated, whereas high serum (20 % FBS) confluent HUVECs have a cilia incidence of 2.1 ± 2.2 % (median ± interquartile range). We report, for the first time, the presence of cilia in the HMEC-1 cell type. HMEC-1s have between 2.2 and 3.5 times greater cilia incidence than HUVECs (*p* < 0.001). HMEC-1s also have shorter cilia compared to HUVECs (3.0 ± 1.0 μm versus 5.1 ± 2.4 μm, at confluence, *p* = 0.003).

**Conclusions:**

We demonstrate that FBS plays a role in determining the prevalence of cilia in HUVECs. In doing so, we highlight the importance of considering a commonly varied parameter (% FBS), in the experimental design. We recommend that future studies examining large blood vessel EC primary cilia use confluent HUVECs grown in high serum medium, as we found these cells to have a higher cilia incidence than low serum media HUVECs. For studies interested in microvasculature EC primary cilia, we recommend using cobblestone HMEC-1s grown in high serum medium, as these cells have a 19.5 ± 6.2 % cilia incidence.

**Electronic supplementary material:**

The online version of this article (doi:10.1186/s13630-015-0020-2) contains supplementary material, which is available to authorized users.

## Background

Endothelial cell (EC) primary cilia are sensors of blood flow-induced mechanical forces [1, 2]. An important component of these forces is fluid-induced wall shear stress (WSS), defined as the drag force (in the flow direction) over the wall area. ECs are particularly sensitive to fluid-induced WSS. For example, ECs are able to detect and respond to WSS variation within seconds, at microscopic spatial resolution [3]. Both low and oscillatory WSS have been implicated as potential causes of cardiovascular diseases such as atherosclerosis [4].

EC primary cilia are thought to be specialised sensors of low and oscillatory WSS due to a number of observations. Primary cilia are able to dynamically alter their length in response to WSS [5], disassemble in response to high WSS (>1.5 Pa) [6], and are typically absent in regions of high WSS [7]. Throughout the vasculature, primary cilia are prevalent in regions that are exposed to low or oscillatory WSS [8–10]. Low WSS regions are also where atherosclerotic lesions typically develop. Furthermore, primary cilia dysfunction has been implicated in the development of a number of cardiovascular disorders including hypertension, the development of aneurysms and Bardet–Biedl syndrome [7]. It has been suggested that the presence of primary cilia represents a restorative attempt by the body to prevent atherosclerosis [11]. Although the precise role of primary cilia in the vasculature remains unknown, we believe that there is increasing evidence to support a role for EC primary cilia in atherogenesis.

Previous studies have used human umbilical vein endothelial cells (HUVECs) to examine primary cilia in the vasculature. Geerts et al. [12] demonstrated that HUVECs cultured in vitro beyond the point of confluence form a ‘cobblestone’ morphology, whereby HUVECs establish cell–cell contacts such as tight junctions. This condition is thought to mimic the in vivo lining of umbilical veins, thus providing an appropriate human cell line model [12]. HUVECs have also been employed to demonstrate cilia disassembly in high fluid shear stress of greater than 1.5 Pa [6]. Beyond cilia studies, HUVECs are a common model used to study the interaction of ECs with fluid-induced WSS. Several studies have shown, using HUVECs, that WSS and WSS gradients affect EC morphology [13], alignment [14, 15] and transcription profile [16]. Furthermore, HUVECs have been used to investigate the mechanisms by which high WSS can lead to athero-protective EC states [17].

The percentage of ciliated cells (cilia incidence) varies greatly across studies that have examined primary cilia in HUVECs. Geerts et al reported a $\sim$30 % cilia incidence in HUVECs in vitro [12], Iomini et al. reported a $\sim$8 % cilia incidence [6], whereas Wheatley et al. reported a 0 % cilia incidence [18]. We hypothesise this is due to the varying conditions in which the cells were cultured. For example, Geerts et al. cultured HUVECs in 2 % foetal bovin serum (FBS) with endothelial cell growth medium-2 (EGM-2) and l-glutamine, whereas Iomini et al. cultured HUVECs in 20 % FBS containing 4 % human serum, vascular endothelial growth factor (VEGF), fibroblast growth factor basic (FGFb) and heparin.

Both growth to confluence and serum starvation are commonly used methods to promote ciliogenesis. This is because primary cilia reabsorb during the early stages of mitosis [19], and re-assemble during exit from the cell cycle. Therefore, confluent cells cultured in low or zero growth-supplemented media are induced into a state of differentiation, thus have increased likelihood of ciliation [20]. In particular, most in vitro studies of cells from many different tissue types usually serum starve cultures for between 24 and 72 h prior to stimulation or analysis [21–25]. Both Geerts et al. and Iomini et al. grew cells to confluence prior to quantifying cilia incidence, but neither Geerts et al. nor Iomini et al. serum-starved HUVECs prior to examining cilia incidence.

Another commonly used EC model is the human microvascular endothelial cell (HMEC-1) line. HMEC-1s were created by immortalising human dermal microvascular ECs with a simian virus 40 large T-antigen plasmid [26]. They have been used to examine the regulatory pathways of EC growth hormones [27], the EC response to inflammatory proteins [28] and the feasibility of using micro-circulation flow chambers to maintain healthy endothelial layers in vitro [29]. While HMEC-1s are a commonly used model for studying the microcirculatory endothelium, primary cilia incidence in HMEC-1s has not yet been examined.

In this study, we aim to determine the effect of cell culture conditions on primary cilia incidence in two different cell types. We examine HUVEC and HMEC-1 cilia incidence in response to (1) the effect of FBS concentration in the media, (2) post-confluence growth to promote cell–cell contact (cobblestone morphology) and (3) serum starvation. In doing so, we aim to explain the variation in reported cilia incidence. Furthermore, we aim to determine optimal cell culture conditions that maximise EC primary cilia incidence, which will be of use to future EC primary cilia studies.

## Methods

### Cell culture

**Fig. 1:**
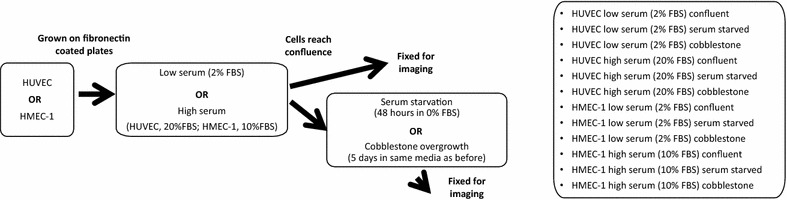
Flow diagram illustrating the cell culture conditions of HUVEC and HMEC-1 used in this study. First, during growth prior to confluence, cells are grown in either low or high serum media treatment. Upon confluence, cells were either fixed or subjected to serum starvation for 48 h or cobblestone treated (grown for an additional 5 days in the corresponding expansion media). All 12 combinations were assessed

Figure 1 illustrates the cell culture work flow and different treatments used in this study. Unless otherwise stated, all materials were obtained from Life Technologies (Carlsbad, CA, USA). HMEC-1s were kindly provided by Dr. Edwin Ades, Mr. Francisco J. Candal (CDC, Atlanta GA, USA) and Dr. Thomas Lawley (Emory University, Atlanta, GA, USA). HUVECs (#C-003-5C) between passages 2 and 4, and HMEC-1s between passages 5 and 7 were seeded at a concentration of 1 × 10^5^ cells/ml (1 × 10^4^ cells/cm^2^) onto either (1) fibronectin-coated 6-well plates (fibronectin, 20 μg/ml, #33016-015) or (2) fibronectin-coated chamber slides (25 μg/ml, #354559, BD, Franklin Lakes, NJ, USA). They were grown to confluence at 37 °C in 5 % CO_2_.

HUVECs and HMEC-1s were grown in either high or low serum media until confluence, at which point they were either fixed for imaging or subjected to one of two post-confluence treatments: (1) serum starvation with a serum-free media for 48 h; (2) growth beyond confluence for 5 days, herein referred to as cobblestone treatment (see Fig. 1).

### Culture media

HUVECs subject to low serum pre-confluence treatment were maintained in M200 media (#M200-500) with low serum growth supplement (#S-003-10, final concentration: FBS, 2 % v/v; hydrocortisone, 1 μg/ml; human epidermal growth factor, 10 ng/ml; basic fibroblast growth factor, 3 ng/ml; and heparin, 10 μg/ml), and penicillin/streptomycin (at 100 U/ml and 100 μg/ml concentration, respectively, #15140122). HUVECs subject to high serum pre-confluence treatment were maintained in identical media formulation with the addition of FBS (#10091148) to result in 20 % v/v final concentration.

HUVECs that were cobblestone treated were maintained in the same media as used pre-confluence. HUVECs subject to serum starvation post-confluence were maintained in media consisting of M200 with penicillin/streptomycin (100 U/ml and 100 μg/ml concentration, respectively).

HMEC-1s subject to low serum pre-confluence treatment were maintained in MCDB131 (#10372019) media with 2 mM l-glutamine (#25030081), 2 % FBS and penicillin/streptomycin (100 U/ml and 100 μg/ml concentration, respectively). HMEC-1s subject to high serum pre-confluence treatment were maintained in identical media formulation with the addition of FBS to result in 10 % v/v final concentration.

HMEC-1s subject to cobblestone post-confluence treatment were maintained in the same media as used pre-confluence. HMEC-1s subject to serum starvation post-confluence were maintained in media consisting MCDB131 (#10372019) media with 2 mM l-glutamine (#25030081) and penicillin/streptomycin (100 U/ml and 100 μg/ml concentration, respectively). Media was refreshed daily.

### Immunofluorescence labelling

Although acetylated $\alpha$-tubulin-based approaches have traditionally been used to visualise the primary cilium using immunofluorescence techniques [6, 10, 12, 30–32], more recently, Caspary et al. have developed an antibody that targets the arl13b protein, a small GTPase protein localised to the axonemal portion of the primary cilium [33]. In this study, we identified cilia using both arl13b antibody and 611b acetylated $\alpha$-tubulin antibody. The nucleus was also stained with 33,258 DNA dye. The staining protocol is as follows: cells were fixed with 4 % paraformaldehyde (#158127-100, Sigma-Aldrich, St Louis, MO, USA) in PBS (#00-3000) for 30 min at 37 °C, then washed with PBS (3 × 5 min). Cells were then permeabilised with triton X-100 (0.5 %, 5 min, #T9284, Sigma-Aldrich), then washed with PBS (3 × 5 min). This was followed by 30-min blocking with goat serum (1:20, #G9023 Sigma-Aldrich) at room temperature.

Rabbit polyclonal arl13b (1:300, #17711-1-AP, Protein Tech, Chicago, IL, USA) was then applied overnight at 4 °C. Cells were then washed with PBS (3 × 10 min), and incubated for 2 h at room temperature with secondary antibody goat anti-rabbit Alexa Fluor 488 (1:500, #A-11008), followed by another PBS wash (3 × 10 min).

Cells were then fixed in 4 % paraformaldehyde, washed in PBS (3 × 5 min), and blocked in goat serum (30 min at room temperature), followed by an overnight incubation in 611b (1:500, #T7451 Sigma-Aldrich). Cells were then incubated for 2 h with secondary antibody goat anti-mouse Alexa Fluor 594 (1:500, #A11005), and then washed with PBS (3 × 5 min). Cells were then stained with Hoechst 33258 (1:1000, #B2883, Sigma-Aldrich) for 5 min at room temperature and washed with PBS (3 × 5 min). Next, coverslips were mounted using ProLong Gold (#P36934). In the case of 6-well plates, coverslips were directly mounted onto the stained cells on the plates. Once cured, the bottom of each well (with coverslip attached) was cut out using a heated scalpel to allow direct imaging.

### Microscopy and image analysis

**Fig. 2:**
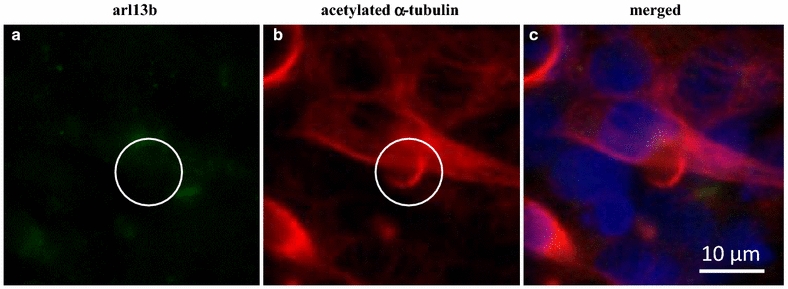
Single staining leads to false positive detection of primary cilia. Arl13b (*green*) and acetylated $\alpha$-tubulin (*red*) labelling in low (2 % FBS) serum HMEC-1, showing a lack of staining with arl13b but a false positive cilium labelled with 611b. Nuclei are labelled with Hoecsht (*blue*)

Cells were imaged using an Olympus FV1000 laser scanning confocal microscope. Diode-pumped 405 nm, argon ion multiline 458 nm and helium neon 543 nm lasers were used in conjunction with appropriate filters to acquire images of the nucleus (Hoechst dye), primary cilium (arl13b bound to goat anti-rabbit Alexa Fluor 488) and acetylated $\alpha$-tubulin (611b bound to goat anti-mouse Alexa Fluor 594), respectively. A 60×/1.35 NA oil immersion lens and sequential excitation with four line averaging were used during image acquisition. Image resolution acquired was 1600 × 1600 pixels, with an XY spatial resolution 0.132 μm/pixel. To avoid bias, images were acquired in a non-overlapping row left-to-right, top-to-bottom, starting from the top left-hand corner of the slide. This was done until 10 images were recorded in every slide. Acquired images were then analysed using Fiji Software (version 1.49m, http://fiji.sc/Welcome) [34]. Every cell in each image was counted by counting total nuclei, using the ‘thresholding tool’, followed by the ‘analyse particles’ tool in Fiji (selecting features greater than 20 μm^2^ in area). This automated method was checked in each image by manually counting. Every cilia in all images was counted. In every experiment in this study (both cell types, high and low serum conditions, regardless of post-confluence treatment), there were instances where primary cilium signal was detected in the 611b acetylated $\alpha$-tubulin channel but not the arl13b cilia GTPase channel (see Fig. 2). These instances were not attributed as actual primary cilium but instead counted separately. There were no observed instances where the opposite occurred: if a primary cilium was detected using arl13b, a positive cilium signal would also be detected in the 611b channel. Cilia length was also determined manually by the same user using the ‘line segmentation’ tool with calibrated images in Fiji. This method had less than 0.2 μm difference compared with z projection method in Fiji, using confocal stacks, indicating cilia were in plane in our coverslipped 2-D culture. Cilia incidence was determined by dividing the number of cilia by the number of nuclei for a given region of interest.

### Statistical analysis

Statistical analysis was performed using R software (version 3.1.2) [35, 36].

Poisson regression was used to compare cilia incidence between populations with differing cell culture conditions. A *p* value of less than or equal to 5 % was considered significant and 95 % confidence intervals were used. Main effects, two-way and three-way interactions were all tested (see Additional file 1 for fitted equations). Models are hierarchical: the two-way interaction model includes main effects and the three-way interaction model includes two-way interactions. The two-way interaction model was used to test for differences in cilia incidence between individual populations.

Cilia length measurements were log-transformed as the data were right-skewed (see Additional file 1). The effect of different cell culture conditions on log (cilia length) was examined using Tukey honest significant difference (Tukey HSD) post hoc tests. A *p* value of less than or equal to 5 % was considered significant.

Images taken from the same slide or well were combined together to represent a single repeat (*N*, number of repeats = 3–5; *n*, number of cilia for each of the 12 conditions ≥13). A total number of 13,270 cells were examined, see Additional file 2 and 3 for the full cilia length and incidence data sets.

## Results

### Cell characteristics

**Fig. 3:**
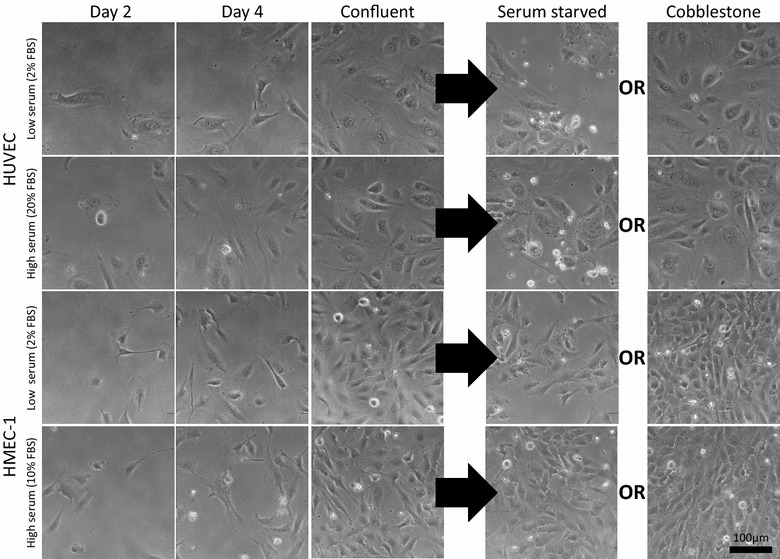
HUVEC and HMEC-1 morphology. Phase contrast images of HUVECs and HMEC-1s cultured on fibronectin-coated plates in low or high serum concentrations. Cells are grown (shown at 2 and 4 days post-seeding) to confluence, and then either fixed for imaging or serum starved (48 hours) or cobblestone treated (5 days further growth)

During the cell expansion phase, the mean time to confluence for HUVECs was 10.0 ± 0.3 days and 7.8 ± 0.5 days in low and high serum conditions, respectively. In contrast, HMEC-1s reached confluence approximately 2–3 days earlier, with a mean time of 7.2 ± 0.7 and 5.1 ± 0.3 days in low and high serum, respectively (Table 1). HUVECs showed a mixed morphology during the expansion phase with a combination of spindle-shaped elongated and flattened orthogonal-shaped cells, whereas HMEC-1 cultures were more consistent, showing spindle-shaped elongated cells throughout the expansion and post-confluent phases. Serum concentration did not appear to alter cell morphology, as shown in Fig. 3. At confluence, both cell types adopted a cobblestone morphology and did not overgrow on top of one another. HUVECs were considerably larger compared to HMEC-1s, with a mean cell area of 510 ± 100 μm^2^ compared to 215 ± 84 μm^2^. During serum starvation, cell number reduced and the confluent layer of cells was lost in both cultures (Fig. 3).

**Table 1:** Mean ± standard deviation time to confluence in HUVECs and HMEC-1s

Cell type	Expansion media	Time to confluence (days)
HUVEC	Low serum (2 % FBS)	10.0 ± 0.3
	High serum (20 % FBS)	7.8 ± 0.5
HMEC-1	Low serum (2 % FBS)	7.2 ± 0.7
	High serum (10 % FBS)	5.1 ± 0.3

### Detection of primary cilia

Using both arl13b and 611b, we observed a total of 728 cilia. We also found 1121 false positive cilia (see Fig. 2).

### Primary cilia incidence

**Fig. 4:**
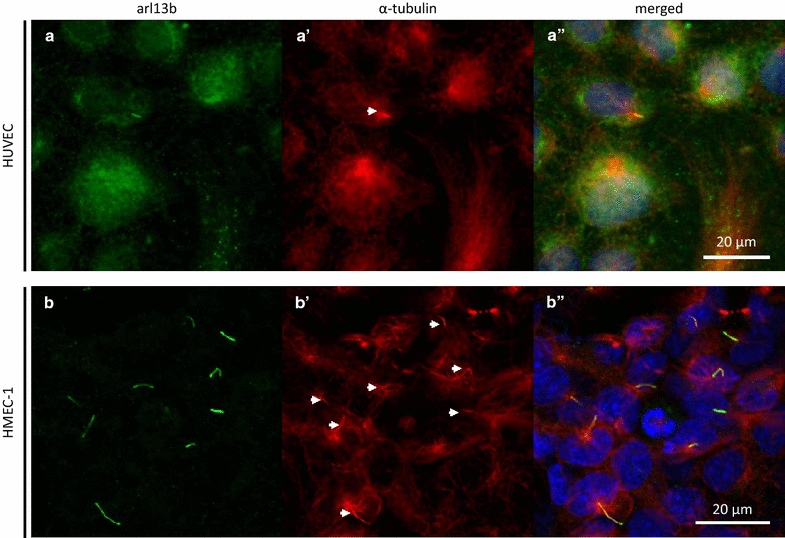
Use of co-labelling to identify primary cilia HUVEC and HMEC-1 primary cilia identification using both axonemal marker arl13b (*a*, *b*) and acetylated $\alpha$-tubulin marker 611b (*a*′, *b*′, *white arrows*). (*a*″, *b*″) Co-localisation of both antibodies indicates presence of cilium. *a*-*a*′) HUVECs grown in high serum (20 % FBS) confluent. *b*, *b*″ HMEC-1s are grown in low serum (2 % FBS) then serum-starved (0 % FBS for 48 h)

Using a combination of arl13b and acetylated $\alpha$-tubulin positive labelling, primary cilia were identified on both HUVECs and HMEC-1s (Fig. 4), with greatest incidence of 19.5 ± 6.2 % (median ± quartile range) in HMEC-1s expanded in high serum conditions followed by culture in high serum media for 5 days.

HUVECs showed low cilia incidence in all conditions, with the maximal incidence of 2.6 ± 3.6 % (median ± quartile range) in the population that was expanded in high serum followed by serum starvation. Expansion of HUVECs in low serum conditions prevented any ciliogenesis at confluence or in either two post-confluence conditions.

Considering all populations together, cell type, serum concentration during expansion and post-confluent condition all had a significant impact on cilia incidence. Based on a 95 % confidence interval, HMEC-1s had between 2.2 and 3.5 times greater cilia incidence than HUVECs (*p* < 0.001). Cells expanded in high serum had between 1.1 and 1.5 times greater cilia incidence than cells expanded in low serum (*p* = 0.0131). Serum-starved cells had between 1.3 and 2.1 times greater cilia incidence than confluent cells (*p* < 0.001). Cobblestone overgrowth had between 2.2 and 3.5 times greater cilia incidence than confluent cells (*p* < 0.001).

**Fig. 5:**
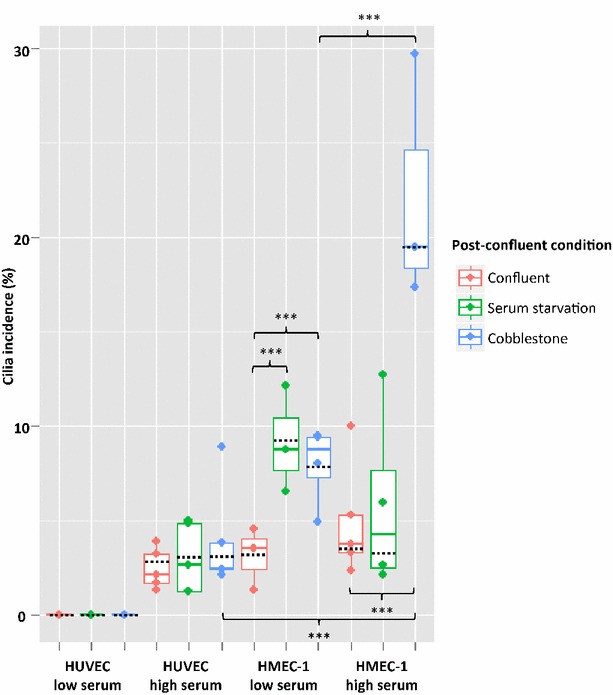
Cilia incidence in HUVEC and HMEC-1. Low serum (2 % FBS) HUVECs did not express any cilia, whereas high serum (20 % FBS) HUVECs expressed cilia in all assessed conditions. High serum (20 % FBS) HUVECs had significantly lower cilia incidence than high serum (10 % FBS) HMEC-1s, in cobblestone population (*p* < 0.001). Cobblestone HMEC-1 cultures in low serum (2 % FBS) had significantly lower cilia incidence than high serum (10 % FBS) HMEC-1s (*p* < 0.001). Cilia incidence in high serum (20 % FBS) HUVECs is not significantly different in either confluent, serum-starved or cobblestone populations. Serum-starved and cobblestone low serum (2 % FBS) HMEC-1s have significantly higher cilia incidence than confluent low serum (2 % FBS) HMEC-1s (*p* < 0.001, for both). Cobblestone high serum HMEC-1s have significantly higher cilia incidence than confluent high serum HMEC-1s (*p* < 0.001). *Boxplots* the median with *upper* and *lower* quartiles, all data points have been plotted. *Black dotted lines* the predicted data point for that culture condition using the two-way interaction model. ***p < 0.001

Inclusion of two-way interaction led to a greater improvement in model fit (see Additional file 1). Observed data versus predicted data from the two-way interaction model are shown in Fig. 5 (dashed lines). There was significant interaction between serum and condition, and cell type and condition, but no significant interaction between cell type and serum levels.

Post-confluent conditions had no significant effect on HUVECs. Cilia incidence significantly increased in cobblestone-treated HMEC-1s compared to confluent HMEC-1s, for low and high serum conditions (Fig. 5*p* < 0.001 and *p* < 0.001, respectively), whereas incidence only increased in serum-starved cultures that had been expanded in low serum (Fig. 5*p* < 0.001). High serum cobblestone HMEC-1 cultures also had a significantly greater incidence than the corresponding low serum condition (Fig. 5*p* < 0.001). HMEC-1s showed a trend of greater cilia incidence compared with HUVECs, with significant differences observed in high serum cobblestone-treated populations (Fig. 5*p* < 0.001). Three-way interaction between cell type, serum level and post-confluent condition was not significant, and this model did not improve the model fit to the observed data, hence was discarded.

### Cilium length

**Fig. 6:**
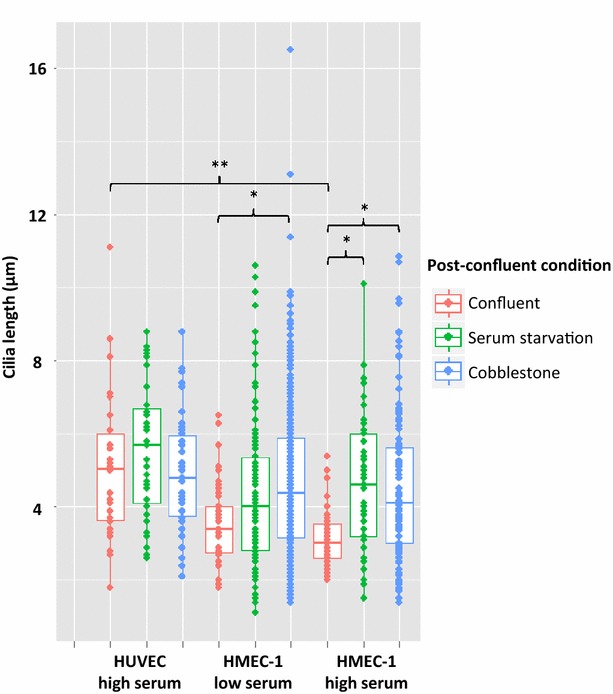
Cilia length of HUVEC and HMEC-1. Post-confluent conditions did not significantly affect HUVEC cilia length. Confluent high serum (20 % FBS) HUVECs have longer cilia than confluent high serum HMEC-1s (10 % FBS), (**p = 0.0032). Cilia length did not significantly differ between HMEC-1s grown in low (2 % FBS) and high (10 %) serum prior to confluence, regardless of post-confluent condition. In low serum HMEC-1s, cobblestone treatment resulted in longer cilia compared to confluent HMEC-1s (*p = 0.0309). In high serum HMEC-1s, both serum starvation (*p = 0.0267) nd cobblestone (*p = 0.0104) post-confluent treatments resulted in longer cilia compared to confluent HMEC-1s. *Boxplot* the median with upper and lower quartiles, all data points have been plotted. Data have a minimum of 13 cilia per condition, from a total of between 3 and 5 experiments

Primary cilia length ranged between 1.8 and 11.1 μm and 1.1 and 16.5 μm in HUVECs and HMEC-1 cells, respectively. However, Fig. 6 shows that confluent HUVECs had significantly longer cilia than confluent HMEC-1s expanded in high serum (*p* = 0.0032). In HMEC-1s expanded in high serum, serum starvation and cobblestone post-confluent treatment resulted in significantly longer cilia compared to confluent HMEC-1s. In HMEC-1s expanded in low serum, this length increase was only observed in cobblestone-treated cells (Fig. 6). See Additional file 1: Table S2 for the data in tabular format.

### Cilia–cilia contact

**Fig. 7:**
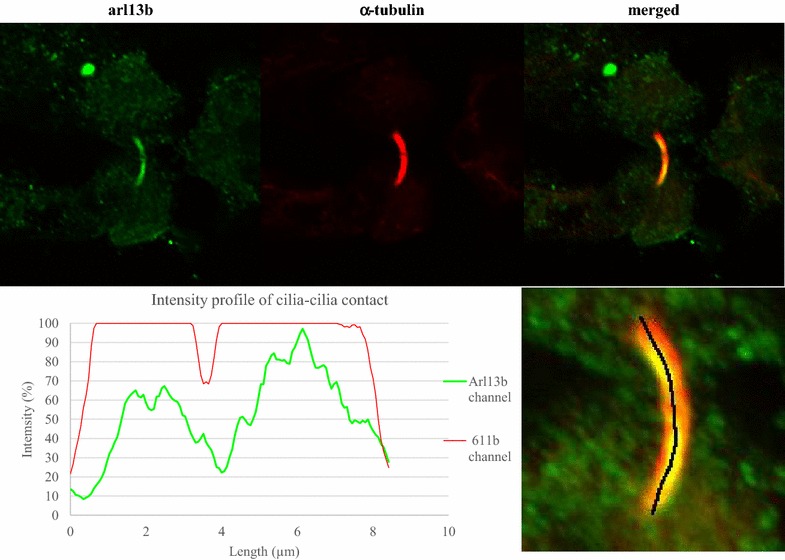
Cilia–cilia contact observed in adjacent ciliated high serum (10 % FBS) HMEC-1s, at confluence. From 39 cilia, 6 formed contacts (3 pairs). The intensity profile shows a reduction in intensity at the same location in both arl13b and 611b channels, indicating two separate cilia, not a single long cilia. The *black line* in RHS indicates the path of the intensity profile. Cilia–cilia contact was not observed in any other culture condition. See Additional file 1: Figure S3 for the other data

In high serum confluent HMEC-1 cells, we observed 6 cilia–cilia contacts from a total of 39 cilia (Fig. 7). Contacts were not observed in high serum HMEC-1s that were serum-starved or grown for 5 days post-confluence. Contacts were also not observed in low serum HMEC-1s, nor in HUVECs. At the point between the two cilia, there is lower signal intensity in both the arl13b and 611b channels, suggesting that the observed contact is not a single long cilia. Bottom right hand panel illustrates the path of the intensity plot (in black).

## Discussion

We investigated culture conditions which had been previously reported in two different HUVEC primary cilium studies conducted by Geerts et al. [12] and Iomini et al. [6], to determine if cilia incidence was due to variations in the culture conditions. Similar passage number, substrate stiffness and media composition were employed. We found that, in HUVECs, low serum concentration prior to confluence abolishes primary cilia, whereas high serum concentration promotes ciliogenesis. HUVEC cilia incidence was not affected by post-confluence treatment. To determine if this serum-dependent incidence occurred in other EC types, we then examined the HMEC-1 cell type. We are able to report that HMEC-1s are ciliated in every treatment we examined. In general, HMEC-1s have a higher cilia incidence than HUVECs, and have increased cilia incidence in response to both serum starvation and cobblestone treatment. We have determined a number of suggestions to aid future EC primary cilium studies which we outline in this section.

Iomini et al. [6] reported 8 % cilia incidence in HUVECs grown to confluence in 20 % FBS under static conditions. This is in reasonable agreement of our findings of 2.1 %. Furthermore, if we use a single acetylated $\alpha$-tubulin-based method to identify the primary cilium, as did Iomini et al., we report a median cilia incidence rate 4.7 %. We observed that low FBS concentration during the expansion phase inhibits the presence of primary cilium in HUVECs. This response is different to that observed by Geerts et al. [12], who measured 30 % cilia incidence in cobblestone (4 days post-confluence) HUVECs grown in a media with 2 % FBS. They visualised cilia using acetylated $\alpha$-tubulin antibody. Using the same visualisation method, we found 8.5 % cilia incidence (see Additional file 1: Table S3). We speculate that this difference may be due to cilia identification criteria. Geerts et al. identified cilia as a red (positive signal in the acetylated $\alpha$-tubulin channel) swab near the nucleus, 2–2.5 μm in length, and surrounded by an acetylated $\alpha$-tubulin positive “cloud” of lower intensity. They identified this cloud as the golgi apparatus (confirmed through double labelling with GM130 golgi marker). While we did not stain for the golgi apparatus, we also detected clouds of acetylated $\alpha$-tubulin in HUVECs (see Fig. 4). Within these clouds, there were bundles of tubulin less than 2.5 μm in length. However, since the vast majority of our double-labelled high serum HUVEC cilia were 4 μm or greater, we did not consider these shorter bundles as cilia in the low serum HUVECs (when carrying out a single label acetylated $\alpha$-tubulin-based identification approach), which may have led to underreporting of cilia incidence compared to the Geerts et al. methodology. It should be noted we did not have a lower limit of cilia length in our identification criteria when using both arl13b and acetylated $\alpha$-tubulin antibody.

FBS is comprised of growth factors, hormones, transport proteins and trace elements. It is added to cell culture media to aid cell growth and proliferation, supplement nutrition, and provide material for extracellular matrix attachment [37, 38]. It is worth noting that the low FBS concentration (2 %) used in this study is the manufacturer’s suggested media formulation for HUVECs (#C-003-5C, Life Technologies). While this concentration is sufficient for healthy HUVEC growth, it was insufficient for ciliogenesis. Further study is needed to determine the specific agents and concentration present in FBS required to promote ciliogenesis in HUVECs. In particular, fibroblast growth factor (FGF) is a candidate media component that has been shown to have an effect on cilia length [39], and is naturally generated at a higher concentration in HMEC-1 than HUVEC [40]. One challenge in determining if FGF (or other growth factors) affect ciliogenesis is that there are already many of these factors present in FBS. The composition of FBS varies between manufacturers, as well as varying between batches from the same manufacturer, and is also affected by season [41, 42]. Using defined media to control for this variation would be a useful extension of this study.

In confluent HMEC-1s, we found that cilia incidence is not significantly affected by serum concentration prior to confluence. We speculate that ciliogenesis in HMEC-1s is a process that is more robust against fluctuations due to variation between batches of FBS as well as between manufacturers, compared to HUVECs. In HMEC-1s, both the post-confluent treatments increased cilia incidence and length. This may be due to a greater proportion of the cells fully differentiating, thus being more likely to express a cilium [21]. Another possibility is that primary cilia play a role in inhibiting growth as part of cell–cell contact inhibition [43]. However, the same was not observed in HUVECs. This ciliogenesis variation between HUVEC and HMEC-1 may be due to the differences between primary cells (HUVEC) and a immortalised cell line (HMEC-1). This difference also accounts for the faster growth in HMEC-1.

In light of our findings, we have some suggestions to aid future EC primary cilium studies. High serum (20 % FBS) HUVECs are a suitable in vitro model for studying primary cilia. We recommend that the HUVECs be grown to confluence (not serum-starved or cobblestone), to reduce the complexity and duration of cell treatment, without affecting cilia incidence or length. Alternatively, low serum (2 % FBS) HUVECs may be an appropriate model for studies that aim to examine cilia-free cells, without having to apply shear flow, or other de-cilation methods such as chloral hydrate [32].

HMEC-1s have significantly higher incidence than HUVECs, reach confluence faster, and are more robust against FBS fluctuations. Hence, they could be a useful alternative model for studying EC primary cilia, provided that a microvascular cell line is physiologically appropriate to the aims of the study. We suggest expansion of HMEC-1s for 5 days post-confluence in high serum media to provide increased cilia incidence and length.

Lastly, we recommend the use of arl13b antibody to identify primary cilia, either solely, or in conjunction with acetylated $\alpha$-tubulin antibodies. We found that use of acetylated $\alpha$-tubulin alone led to a higher estimated cilia incidence compared to double-labelled co-localised imaging using arl13b and 611b acetylated $\alpha$-tubulin antibody. This is expected, as acetylated $\alpha$-tubulin also labels tubulin within the cytoplasm, which can lead to false positives if large bundles are present. In our co-localised imaging, we did not observe a primary cilium using arl13b antibody that did not also have a corresponding signal from the acetylated $\alpha$-tubulin channel. Thus, we suggest that the arl13b antibody may be sufficient on its own to identify primary cilium.

Direct physical contact between primary cilia has been observed in kidney and liver mammalian cells [44]. In these cell types, cilia–cilia connection is known to be stable, persisting for hours, and resists breaking when perturbed with proteases or chemical agents. However, the function of these cilia–cilia connections is unknown. To our knowledge, our study is the first to show evidence of primary cilia–cilia connection in ECs. Further analysis is needed with basal-body co-label imaging to confirm the connection, in conjunction with contact disruption to determine the stability of these connections over time.

## Conclusions

In summary, HUVEC primary cilia incidence is dependent on culture conditions. In particular, high serum (20 % FBS) concentration prompts ciliogenesis, whereas low serum (2 % FBS) concentration inhibits cilia. Furthermore, we report for the first time, primary cilia in HMEC-1 cells. Finally, we have made a number of suggestions to aid future studies on EC primary cilia.

## Additional files

**Additional file 1.** Tabular representation of results, and statistical analysis of cilia incidence and length. Multiple comparison analysis was performed on cilia length and cilia incidence data. This study had 2×2×3 factor setup, where factors were cell type (HUVEC, HMEC-1), serum levels during expansion (low, high) and post-confluent condition (confluent, serum starvation, cobblestone).
**Additional file 2.** Data set of primary cilium length in response to different culture conditions (n = 728). Headings are: CELL TYPE (HUVEC or HMEC-1, SERUM (high or low fetal bovine serum during expansion), CONDITION (the post-confluent condition applied to the cells: confluent, serum starvation or cobblestone, UNIT (the repeat number), NUMBER OF CELLS (total number of cells examined from the unit), NUMBER OF FALSE POSITIVES (number of false positive cilia), NUMBER OF CILIA (total number of cilia) and LENGTH (in microns) of the cilia.
**Additional file 3.** Data set of primary cilium incidence in response to different culture conditions. Headings are identical to additional file 2, except LENGTH is replaced by NUMBER OF CILIA/NUMBER OF CELLS. PERCENTAGE is this value × 100%.

## Abbreviations

EC: endothelial cell
EGM-2: endothelial cell growth medium-2
FBS: foetal bovine serum
FGF: fibroblast growth factor
FGFb: fibroblast growth factor basic
HMEC-1: human microvascular endothelial cell
HUVEC: human umbilical vein endothelial cell
VEGF: vascular endothelial growth factor
WSS: wall shear stress
