# Supplementary material for: Culture and detection of primary cilia in endothelial cell models
Source: Cilia. 2015 Sep 30;4:11. doi: 10.1186/s13630-015-0020-2 (PMC4590708; doi:10.1186/s13630-015-0020-2)
Supplement: Supplementary file 1 — Additional file 1. Tabular representation of results, and statistical analysis of cilia incidence and length. Multiple comparison analysis was performed on cilia length and cilia incidence data. This study had 2×2×3 factor setup, where factors were cell type (HUVEC, HMEC-1), serum levels during expansion (low, high) and post-confluent condition (confluent, serum starvation, cobblestone). [file 13630_2015_20_MOESM1_ESM.pdf]

Results

Table S1 Endothelial cell primary cilia incidence of HUVEC and HMEC-1s in various cell culture conditions. HUVECs grown in high serum (20% FBS) have higher cilia incidence than HUVECs grown in low serum (2% FBS). Post-confluence treatment did not significantly affect HUVEC cilia incidence. HMEC-1s have higher cilia incidence than HUVECs. Following cobblestone treatment high serum (10% FBS) HMEC-1s showed the highest cilia incidence of any of the assessed conditions. Data represents the median ± quartile range from 3-5 experiments.

Cell type	Expansion media	Cilia incidence (%)		
		Confluence	Serum starvation (48 h, 0% FBS)	Cobblestone growth
HUVEC	Low serum (2% FBS)	0.0 ± 0.0%	0.0 ± 0.0%	0.0 ± 0.0%
	High serum (20% FBS)	2.1 ± 2.2%	2.6 ± 3.6%	2.4 ± 1.4%
HMEC-1	Low serum (2% FBS)	3.5 ± 1.6%	8.7 ± 2.8%	8.7 ± 2.2%
	High serum (10% FBS)	3.7 ± 2.0%	4.3 ± 5.1%	19.5 ± 6.2%

Table S2 Cilia length in HUVEC and HMEC-1. Data represents the median ± quartile range from 3-5 experiments.

Cell type	Pre-confluence	Cilia length (μm)		
		Confluence	Serum starvation (48 h, 0% FBS)	Cobblestone growth
HUVEC	Low serum (2% FBS)	n/a	n/a	n/a
	High serum (20% FBS)	5.1 ± 2.4	5.7 ± 2.6	4.8 ± 2.2
HMEC-1	low serum (2% FBS)	3.4 ± 1.3	4.0 ± 2.5	4.4 ± 2.7
	high serum (10% FBS)	3.0 ± 1.0	4.6 ± 2.8	4.1 ± 2.6

Table S3 Measuring primary cilia incidence is dependent on the cilia identification method. This table illustrates cilia incidence as identified solely by acetylated α-tubulin antibody. This resulted in a higher cilia incidence compared to that obtained using both tubulin and arl13b antibody colocalisation (see Table S1). Data represents the median ± quartile range from 3-5 experiments.

Cell type	Pre-confluence	Cilia incidence detected by acetylated α-tubulin antibody (%)		
		Confluence	Serum starvation (48 h, 0% FBS)	Cobblestone growth
HUVEC	Low serum (2% FBS)	7.1 ± 4.0%	4.8 ± 7.5%	11.4 ± 5.3%
	High serum (20% FBS)	4.7 ± 2.4%	6.1 ± 1.3%	7.7 ± 2.3%
HMEC-1	low serum (2% FBS)	5.4 ± 1.8%	14.4 ± 2.2%	10.8 ± 2.5%
	high serum (10% FBS)	6.1 ± 5.1%	7.3 ± 3.2%	22.0 ± 6.1%

Statistical analysis

Multiple comparison analysis was performed on cilia length and cilia incidence data. This study had 2x2x3 factor setup, where factors were cell type (HUVEC, HMEC-1), serum levels during expansion (low, high) and post-confluent condition (confluent, serum starvation, cobblestone).

Primary cilia incidence

Primary cilia incidence data was right-skewed (Figure S1). Cilia incidence was defined as $n_{cilia}/n_{cells} \times 100\%$, where n_{cilia} is the number of cilia and n_{cells} is the number of cells. Hence, number of cilia is a count variable (integer) that is dependent on the number of cells counted (extensive data). Poisson regression is the most

appropriate choice for analysing this type of data [1] [2]. Cilia incidence was analysed using Poisson regression with a log link function offset by the number of cells.

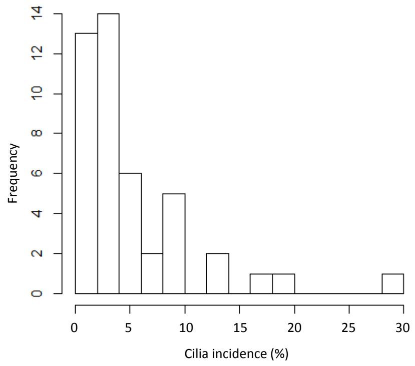

Figure S1 Histogram of cilia incidence (%). Data is right-skewed.

The fitted main effect Poisson regression model is described by the following equation:

$$log\Big(\frac{number\ of\ cilia}{number\ of\ cells}\Big) = B_0 + B_1 Cell\ type + B_2 Serum + B_3 Condition$$

For the 3 factors, the reference levels are HUVEC, low serum during expansion and confluence. The coefficients B_{0-n} refer to the difference in $\log(n_{cilia}/n_{cells})$ changing that particular categorical variable with respect to the reference variable. For instance, if $B_1 = 3$, we would expect $\log(n_{cilia}/n_{cells})$ to increase by 3 if cell type is changed from HUVEC to HMEC-1 (all other factors being held constant). Table S4 shows the main effect model estimates for B_{0-n}. From these estimates and the standard error, 95% confidence intervals can be calculated. These are presented in the results section of the main paper.

Table S4 Poisson regression of main effects.

Parameter	Estimate B_{0-n}	Std. Error	z value	Pr $> \|z\|$
Intercept	-4.44	0.17	-26.21	< 0.001
HMEC-1	1.01	0.12	8.2	< 0.001
high serum	0.23	0.09	2.48	0.0131
cobblestone	0.91	0.11	8.19	< 0.001
serum starvation	0.52	0.12	4.25	< 0.001

The two-way interaction model is described by the following equation:

$$log\Big(\frac{number\ of\ cilia}{number\ of\ cells}\Big) = B_0 + B_1 Cell\ type + B_2 Serum + B_3 Condition$$
$$+ B_4 Cell\ type : Serum + B_5 Cell\ type : Condition + B_6 Serum : Condition$$

[1] Coxe, S., West S.G., Aiken, L.S.: The analysis of count data: A gentle introduction to poisson regression and its alternatives. Journal of personality assessment **91**(2), 121–136 (2009).
[2] Winkelmann, R.: Econometric Analysis of Count Data. Springer, New York, USA (2013)

This model has the same reference levels as main effect model. Inclusion of two-way interactions changes interpretation of B_1. B_1 now refers to effect of cell type at the reference case for serum and condition (i.e. when serum and condition both equal zero) . Hence the two-way interaction model is used to compare individual populations, by altering the reference levels (see Figure 4 of main paper, and Table ??).

Table S5 Significant differences in cilia incidence between different populations, as estimated using Poisson regression two-way interaction model. For brevity only significant interactions are included. Estimate of 0.86 indicates that low cobblestone HMEC-1 have a factor of $e^{0.86}$= 2.36 greater cilia incidence than low confluent HMEC-1s.

Population 1	Population 2	Estimate B_n	Std. Error	z value	Pr > $\|z\|$
low confluent HMEC-1	low cobblestone HMEC-1	0.86	0.16	5.27	< 0.001
high confluent HMEC-1	high cobblestone HMEC-1	1.74	0.19	9.31	< 0.001
low confluent HMEC-1	low serum starved HMEC-1	1.11	0.18	6.137	< 0.001
low cobblestone HMEC-1	high cobblestone HMEC-1	0.99	0.11	8.70	< 0.001
high cobblestone HUVEC	high cobblestone HMEC-1	1.9	0.18	10.39	< 0.001

The three-way interaction model is described by the following equation:

$$log\Big(\frac{number\ of\ cilia}{number\ of\ cells}\Big) = B_0 + B_1 Cell\ type + B_2 Serum + B_3 Condition$$
$$+ B_4 Cell\ type : Serum\ +\ B_5 Cell\ type : Condition\ +\ B_6 Serum : Condition$$
$$+\ B_7 Serum : Condition : Cell\ type$$

Table S6 shows how well each of the models fits the data. Of the three models examined, the two-way interaction model has the best fit.

Table S6 Criteria for Assessing Goodness of Fit of each model. AIC is Akaike information criteria.

Model	Degrees of Freedom	Residual Deviance	Residual Deviance/DF	AIC
One-way interaction	40	195.56	4.89	363.68
Two-way interaction	35	78.23	2.24	256.34
Three-way interaction	33	78.229	2.37	260.34

Three-way interaction between cell type, serum level and post-confluent condition was not significant, and this model did not improve the model fit to the observed data, hence was discarded.

Cilia length

Cilia length is not normally distributed, and is right-skewed (Figure S2). A log transform was applied to our data, which resulted in a normal distribution of aggregate log(cilia length) as well as normal distribution in each of the 9 ciliated populations (HUVEC high confluent, serum starved, cobblestone; HMEC-1 low confluent, serum starved, cobblestone; HMEC-1 high confluent, serum starved, cobblestone).

A three-way ANOVA model with interaction was used to analyse log(cilia length). Analysis was performed using R software (version 3.1.2).

Serum levels during expansion have no significant effect on cilium length (Serum, p = 0.35). Cell type has an effect (p = 2.4e-5), as does post-confluence condition (p = 1.19e-3). There is no significant interaction between cell type and condition

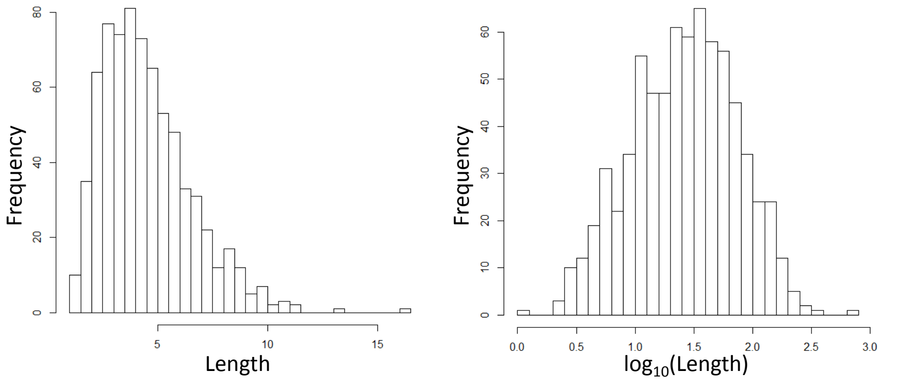

Figure S2 Histogram of cilia length prior and post log transform. Non-transformed data is right-skewed (Shapiro-Wilk test, $p <$ 2.2e-16). Transformed data is normally distributed (Shapiro-Wilk test, $p =$ 0.0516).

Table S7 Three-way ANOVA of log(cilia length). The factors included in the ANOVA were cell type, serum during cell expansion, and post-confluent condition. * indicates significance at 0.05 level, ** at 0.01 level and *** at 0.001 level). Interaction between serum and cell type cannot be considered because there is no data for low serum HUVEC cilia length. Similarly three-way interaction is not considered.

	Degrees of Freedom	Sum of Squares	Mean of Squares	F value	Pr(>F)
Cell type	1	3.38	3.38	18.10	2.38e-5 ***
Serum	1	0.16	0.16	0.88	0.35
Condition	2	3.41	1.706	9.149	1.19e-3 ***
Cell type:Condition	2	0.98	0.49	2.62	0.07
Serum:Condition	2	0.47	0.24	1.27	0.28
Residuals	719	134.10	0.187		

($p = 0.074$), nor between serum and condition ($p = 0.28$). Tukey honest significant difference post-hoc tests were used to determine where the significant interactions occurred, and the results of this analysis are presented in the main paper.

Cilia contact

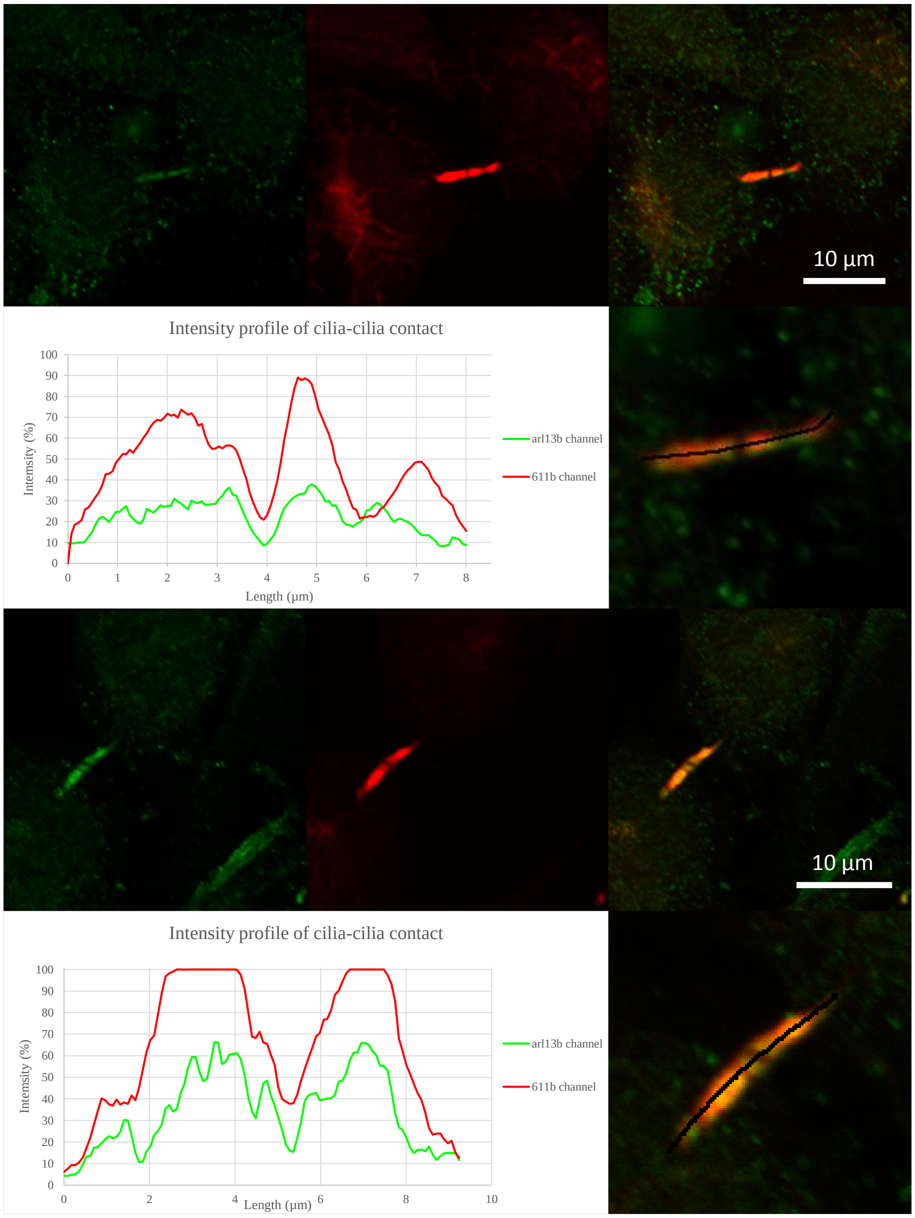

Figure S3 Additional 2 pairs of cilia—cilia contact observed in adjacent ciliated high serum (10% FBS) HMEC-1s, at confluence. From 39 cilia, 6 formed contacts (3 pairs). The intensity profile shows a reduction in intensity at the same location in both arl13b and 611b channels, indicating two separate cilia. The black line in RHS indicates the path of the intensity profile.
